# Distractor Interference during a Choice Limb Reaching Task

**DOI:** 10.1371/journal.pone.0085961

**Published:** 2014-01-17

**Authors:** Matthew Ray, Daniel Weeks, Timothy N. Welsh

**Affiliations:** 1 Faculty of Kinesiology and Physical Education, University of Toronto, Toronto, Ontario, Canada; 2 Department of Psychology, University of Lethbridge, Lethbridge, Alberta, Canada; 3 Centre for Motor Control, University of Toronto, Toronto, Ontario, Canada; University of Reading, United Kingdom

## Abstract

According to action-centered models of attention, the patterns of distractor interference that emerge in selective reaching tasks are related to the time and effort required to resolve a race for activation between competing target and non-target response producing processes. Previous studies have only used unimanual aiming tasks and, as such, only examined the effects of competition that occurs within a limb. The results of studies using unimanual aiming movements often reveal an “ipsilateral effect” - distractors on the same side of space as the effector cause greater interference than distractors on the opposite side of space. The cost of the competition when response selection is between the limbs has yet to be addressed. Participants in the present study executed reaching movements to 1 of 4 (2 left, 2 right) possible target locations with and without a distractor. Participants made ipsilateral reaches (left hand to left targets, right hand to right targets). In contrast to studies using unimanual aiming movements, a “contralateral effect” was observed; distractors affording responses for the other hand (in contralateral space) caused more interference than distractors affording responses for the same hand. The findings from the present research demonstrate that when certain portions of response planning must be resolved prior to response initiation, distractors that code for that dimension cause the greatest interference.

## Introduction

Attention is often characterized as the allocation of cognitive resources to the processing of a specific subset of available information. In the context of goal-directed action, attention is critical for acquiring and accessing sensory information that will support action planning and control [1]. Therefore, it logically follows that attention would be influenced by the needs of our motor system. The tight link between the processes of action and attention and other cognitive processes is the central premise of pre-motor [2] and action-centered [3] models and theories of attention, and the theory of event coding [4].

In the seminal paper on action-centered attention by Tipper et al. [3], the authors used an innovative experimental design that allowed them to test between several different models of attentional coding during goal-directed aiming movements. Specifically, they asked participants to execute goal-directed movements to targets that were presented on a rectangular board with nine target locations arranged in a 3 by 3 grid. One red and one yellow light-emitting diode (LED) was located under each potential target location. The illumination of the red LED indicated the target location and the illumination of the yellow LED (on the majority of the trials) indicated the distractor location. Participants were asked to aim to and touch the target location and ignore the yellow distractor LED. To test the different models of attentional coding in this task, they analyzed the patterns of distractor interference. The measure of distractor interference was the difference in total response time (the sum of reaction time [RT] and movement time [MT]) between no-distractor and distractor-present trials. It was hypothesized that the magnitude of the increase in total response time between no-distractor and distractor-present trials (i.e., distractor interference) represented the time it took for the conflict between competing responses to be resolved.

Two key patterns of distractor interference were reported by Tipper et al. [3]. First, when participants reached to targets in the middle column, distractors that were ipsilateral to the anatomical origin of the effector caused more interference than distractors in contralateral space [the ipsilateral effect]. Second, when participants reached to targets in the middle row, distractors that appeared in the row nearest to the starting position of the hand (proximal row) caused more interference than distractors farther from the starting position of the hand (distal row) [the proximity-to-hand effect]. These patterns of distractor interference were hypothesized to emerge because of the relative salience and efficiency of the competing responses that raced for activation (see [5] for a review). That is, it was hypothesized that the capture of attention by the stimuli automatically activated response-producing processes that competed within the motor system [6] and the amount of interference reflected the time and effort required to eliminate the distractor response, allowing the target response to emerge. When the non-target response is less salient or less efficient to plan and execute than the target (e.g., a non-target movement into contralateral space vs. a target movement into ipsilateral space), little time and effort is required to allow the target response to emerge. On the other hand, when the non-target response is more salient and efficient to plan and execute than the target response (e.g., a non-target movement into ipsilateral space vs. a target movement into contralateral space), then more time and effort is required to prevent the non-target response from emerging, thus leading to larger interference effects.

Since Tipper et al.'s [3] original study, other researchers have provided further insight into the patterns of distractor interference that emerge when competing responses vary in efficiency, direction, amplitude, and spatial distribution [7]–[10]. In addition, several studies have revealed that the presence of a distractor can affect the components of RT and MT in different ways for different individuals (e.g., [8]), and even influence the kinematics of actions in the absence of temporal effects (e.g., [11], [12]). Overall, the results of these studies are generally consistent with the main premise of action-centred attention and have shed new light on the interactions between the processes of action planning and attention. One dimension of response selection that is yet to-be-explored in a distractor interference context is limb selection. All previous studies of action-centered attention using a distractor interference approach (but see [13]) have utilized tasks where only a single effector is required; thus, limb selection was eliminated from the response planning process. In daily interactions, it is often the case that a motor task has response alternatives and the actor needs to determine which limb to respond with. Therefore, the specific focus of the current study was to investigate how response selection between the limbs would influence patterns of distractor interference in a selective reaching task.

There is reason to believe that selection across the limbs may cause as much, if not more, interference than selection within a limb. This pattern could emerge because, despite the fact that competing responses are predominantly represented in (and ultimately issued from) separate brain hemispheres, selection of the responding limb may present a greater challenge to response planning than selection of other movement parameters such as direction and amplitude. Evidence for this possibility was most elegantly provided by Rosenbaum [14] who used a movement precue paradigm to investigate how advance information related to different movement planning dimensions influenced reaction time (RT). Participants in this study performed reaching movements to one of eight targets. The target array consisted of four potential target locations for each hand, with two on either side of the hand, one closer to the hand and one farther from the hand. Thus, to acquire the target, the participant had to select the correct hand, direction, and amplitude. Prior to the onset of the target stimulus, cues were presented that provided either: all information about the upcoming target location (identified the target specifically), partial information about the potential target location (information for one or two of the stimulus dimensions), or no information at all.

One key finding from the Rosenbaum [14] study was that when limb selection was not cued (i.e., the responding limb was unknown) there were larger increases in RT in comparison to when movement direction and amplitude were not cued. In other words, there was a greater cost for not knowing the responding limb in comparison to not knowing movement direction or amplitude. In addition, the RT benefits generated from prior knowledge of direction and amplitude were only realized when the responding limb was known. Based on this pattern of results, it was suggested that people benefit the most from prior knowledge of which limb will be used in comparison to knowing the movement amplitude or direction. What these findings may indicate is that uncertainty related to limb selection must be resolved prior to movement initiation (reflected in increased RTs), unlike with direction or amplitude that can be corrected after movement initiation (which may lead to increased MTs). Overall, the key conclusion of this work is that extra time and care must be taken during response planning when the limb to be used is not specified in advance.

The influence of limb selection on response planning has interesting implications concerning the response competition that typically occurs between targets and distractors in choice reaching tasks. Specifically, the competition between responses for different limbs may need to be fully resolved prior to movement onset, whereas the resolution of response competition within a limb may be resolved during the movement itself with little-or-no temporal cost [11], [12]. Therefore, it is possible that there may be an increased RT cost (interference effect) when target and distractor stimuli activate competing responses in different limbs (a contralateral interference effect) in comparison to target and distractor stimuli that activate competing responses within a limb (ipsilateral interference effect).

To assess the patterns of interference that emerge when distractors and targets activate competing responses within a limb and between different limbs, participants in the present study reached to targets presented in one of four potential target locations that were arranged in a 2×2 grid. Two target locations were on the left side of space and two target locations were on the right side of space. Participants were instructed to respond to targets on the left side of space with the left hand and to targets on the right side of space with the right hand. The target was presented alone on 25% of the trials and a distractor was randomly presented at one of the other locations on the remaining 75% of the trials. When the distractor was present, it could appear on the same side of space as the target (requiring within-limb selection) or on the opposite side of space from the target (requiring between-limb selection). Of critical interest to the current study, were the relative magnitudes of the interference effects that were generated by distractors that were ipsilateral and contralateral to the target.

Several possible patterns of interference could emerge under these conditions. First, it could be that interference effects will be larger for distractors presented on the same side of space as the target than for distractors on the opposite side of space (i.e., an ipsilateral effect; [3]). Such a pattern of distractor interference would suggest that response competition within a limb-hemisphere system requires more time and effort to resolve than response competition between limb-hemisphere systems. Second, it could be that interference effects will be larger for distractors presented on the opposite side of space from the target than for distractors on the same side of space. This pattern of interference would be consistent with the findings of Rosenbaum [14] who found that people benefit most from prior knowledge (precue information) of the limb to be used than prior knowledge about movement direction or amplitude. Finally, it could be that all distractors on both sides of space generate similar levels of interference. This final pattern would suggest that response selection within a limb requires as much time and effort to resolve as competition between limbs.

## Methods

### Participants

Twelve participants (4 male, 8 female; age range 18–25 years old) were recruited from the University of Calgary community. Participants self-reported their handedness (10 right handed, 2 left handed). Participants provided written informed consent and were given verbal instructions about the task. The study consisted of one experimental session that lasted approximately 45 minutes, including optional breaks, and participants were financially compensated for their time. The procedures of the study complied with the ethical standards of the 1964 Declaration of Helsinki regarding the treatment of human participants in research and were approved by the University of Calgary Research Ethics Board.

### Apparatus, Stimulus, and Task

**Figure 1:**
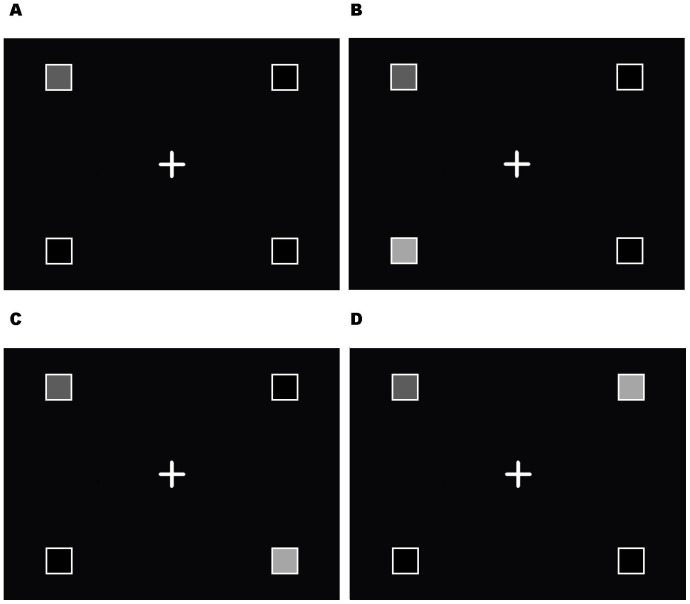
Stimulus displays. Example of stimuli and display for the 4 conditions: a) no distractor condition, b) distractor on the same side of space-different height, c) distractor on the opposite side of space-different height, and d) distractor on the opposite side of space-same height. Dark grey squares represent the target location and light grey squares represent distractor location.

Participants sat on a chair in front of a table. On the table, there was a 19-inch flat screen Viewsonic LCD monitor that was used for stimulus presentation. The monitor was oriented perpendicularly to the table top. There was a fixation cross (3 cm×3 cm) that was displayed in the centre of the monitor. There were four possible target locations displayed on a black background. Each target location was an open white square placeholder (3 cm×3 cm) that was placed in a quadrant of the presentation screen (see Figure 1). The distance between the top row and the bottom row of placeholders was 21 cm and the distance between the right and left row of placeholders was 24 cm. To indicate which locations were the target and the distractor on any given trial, the insides of the placeholder turned red or green, respectively.

There were two home positions marked on the table with a 1 cm×1 cm piece of tape, one for the right hand and one for the left hand. The left hand home position was aligned with the target locations on the left side of screen while the right hand home position was aligned with the target locations on the right side of space. Both home positions were 30 cm from the monitor. An Intel Pentium 3.00 GHz computer was used to run the custom E-Prime V1.1 software that controlled the stimulus events and the initiation of the data collection.

### Procedure

Participants were instructed to make reaching movements, as quickly and accurately as possible, to the red target location and ignore the presence of an irrelevant green distractor when present. Distractors appeared on 75% of the trials and the target was presented alone on the remaining 25% of the trials. If the target appeared at one of the two target locations on the left side of space, then participants reached with their left hand and if the target appeared on the right side of space, then they reached with their right hand.

Each trial began with the participant's right index finger at the right home position and their left index finger at the left home position. A fixation screen, that included the four empty placeholders and the fixation cross, was presented first. The fixation screen was maintained for a random variable foreperiod (1000–3000 ms; in 250 ms increments). Following the variable foreperiod, the target would appear at one of the four target locations with or without a distractor at a different location. When the target appeared, participants reached out and touched the target on the screen with their index finger. There were 4 possible target-distractor combinations: 1) no distractor (target-alone) condition; 2) distractor same side of space-different height; 3) distractor opposite side of space-same height; and, 4) distractor opposite side of space-different height (see Figure 1). The presentation of the trials was randomized and each block included an equal number of trials for each condition at each placeholder. There were 16 familiarization trials at the start of the session (1 trial for each of the 4 conditions at each location) presented in a random order. The experimental phase consisted of a total of 192 trials divided into 6 blocks of 32 trials each (2 trials of each of the 16 trial types presented in a random order).

### Data Collection and Analysis

Data were collected using an active marker system (Visualeyez 3300, PhoeniX Technologies Incorporated). A light emitting diode (LED) was fixed to the fingernails of the participants' right and left index fingers using tape. The location of the LED was recorded at 500 Hz for a 2 s period that began at the onset of the target/distractor array. The data recorded by the camera were filtered using a dual-pass Butterworth filter (2^nd^ order) with a cut-off frequency of 10 Hz. The filtered displacement values were then differentiated twice to obtain instantaneous velocity and acceleration values. Custom software was used to identify the dependent variables. RT was identified as the time interval from stimulus onset to the moment the resultant movement velocity surpassed 30 mm/s and remained there for more than 70 ms. MT was defined as the time interval from the onset of the movement (end of the RT period) until movement velocity in the primary direction (y-axis) fell below 30 mm/s and remained there for 70 ms.

Due to recording errors (loss of LED from the view of the camera), data from 4 of the participants were excluded from the analysis (i.e., 8 participants were included in the final analysis). There were no movements executed to a distractor location (i.e., endpoint errors). A multi-step outlier procedure was applied to all RT and MT data. First, the data for all trials with RTs that were less than 100 ms were eliminated because RTs of this short an interval likely reflect anticipation of the stimuli or artifacts/errors of the analysis program. Subsequently, all data points that were 3 standard deviations greater than the mean value for each participant for a specific condition were eliminated because these data represent trials in which the participant was not paying full attention to the task or made an error, or an artifact/error of the analysis program. This procedure was run twice, once for RTs and then again for MTs. In total, these three procedures resulted in the elimination of 3.6% of the data. Mean RTs and MTs were calculated and then analyzed using a 2 (Hand: right, left) ×4 (Condition: no distractor, distractor same side of space-different height, distractor opposite side of space-different height, and distractor opposite side of space-same height) repeated measures ANOVA. Corrections for sphericity were performed using the Huynh-Feldt procedure (though none were necessary). Post hoc analysis of all significant effects involving more than 2 means was conducted using Tukey's *HSD*. Alpha was set at 0.05 for all analyses.

## Results

### Reaction Time

**Figure 2:**
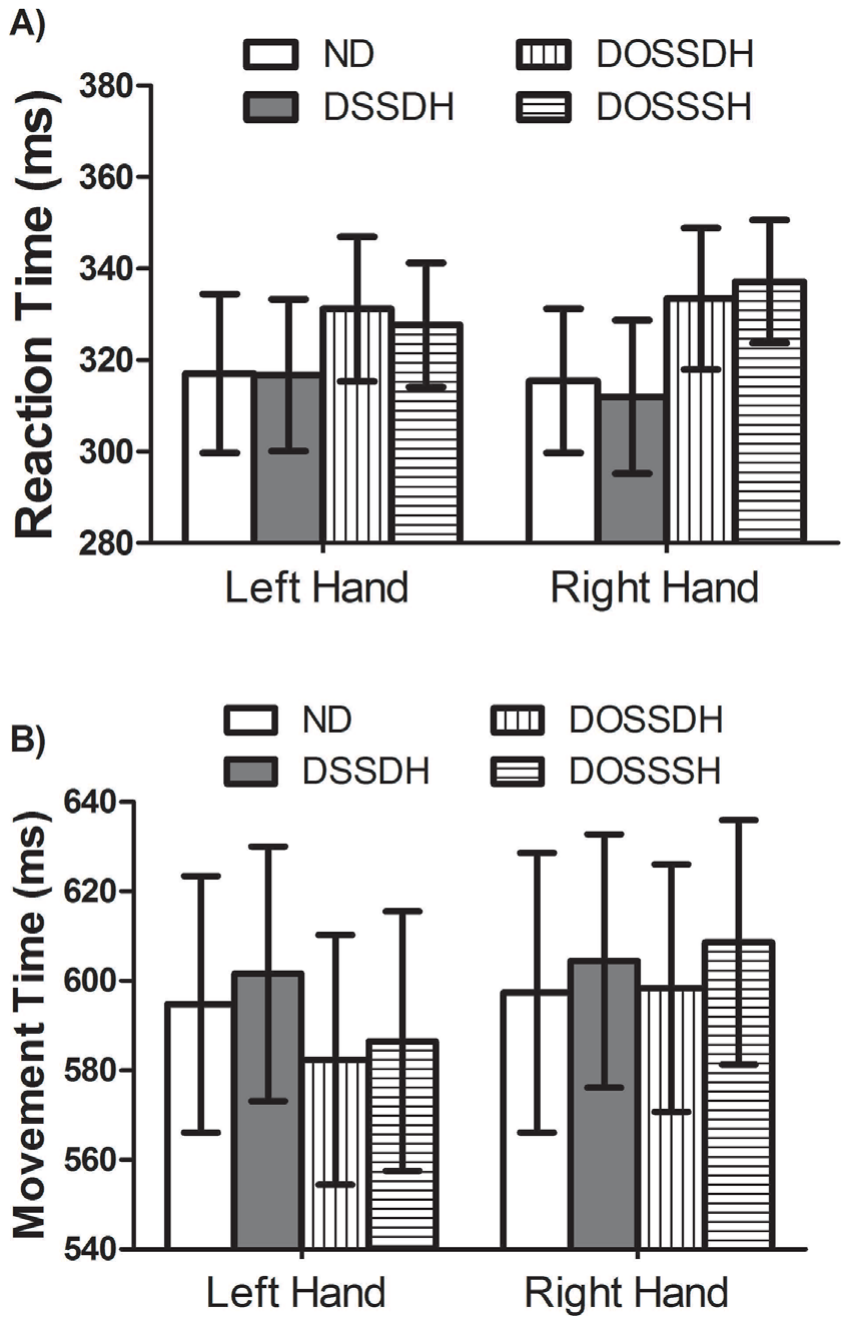
Reaction times (A) and Movement Times (B) during a limb selection reaching task. Reaction times and movement times (in ms) for the left and right hand in the no distractor (ND – open white bars), distractor same side of space-different height (DSSDH – grey bars), distractor opposite side of space-different height (DOSSDH – bars with filled with vertical lines), and distractor opposite side of space-same height (DOSSSH – bars with filled with horizontal lines). SEM bars are shown.

The analysis revealed a significant main effect of Condition, *F*(3, 21) = 10.10, *p*<0.005. Post hoc testing revealed that RTs in the distractor-opposite side of space-different height (*M* = 332 ms, *SD* = 37.4) and the distractor-opposite side of space-same height (*M* = 332 ms, *SD* = 43.6) conditions were significantly longer than the RTs for distractor-same side of space-different height (*M* = 314 ms, *SD* = 45.4) and the no distractor (*M* = 316 ms, *SD* = 45.2) conditions. RTs for the no distractor and the distractor-same side of space-different height conditions were not statistically different. Finally, there was no significant main effect of Hand, *F*(1, 7) = 0.08, *p*>0.79, and the Hand by Condition interaction was not significant, *F*(3, 21) = 0.94, *p*>0.42 (see Figure 2a).

### Movement Time

The main effects of Hand, *F*(1, 7) = 2.39, *p*>0.15, and Condition, *F*(3, 21) = 2.64, *p*>0.1, were not significant. There was, however, a significant interaction between Hand and Condition, *F*(3, 21) = 5.31, *p*<0.05. Post hoc testing of the interaction revealed that when participants used their left hand, MTs in the distractor-opposite side of space-same height (*M* = 582 ms, *SD* = 78.9) were significantly shorter than both the no distractor (*M* = 595 ms, *SD* = 81.1) and the distractor-same side of space-different height (*M* = 602 ms, *SD* = 80.6) conditions. In addition, the distractor-opposite side of space-different height condition (*M* = 586 ms, *SD* = 82.1) was significantly shorter than distractor-same side of space-different height condition (*M* = 602 ms, *SD* = 80.6). The rest of the MTs, including all MTs for the right hand, were not statistically significant from one another (Figure 2b).

### Reaction Time and Movement Time Tradeoff

To determine if a statistically reliable difference in the pattern of RT/MT trade-offs was present across the hand and target conditions, we conducted an additional analysis in which each participant's RT and MT on each trial were correlated within each condition. These r values were then transformed to z scores using Fisher's formula and submitted to two analyses. Although there appears to be a modest overall speed-accuracy (RT/MT) trade-off because the grand mean z score (i.e., averaged across all conditions) of -0.147 was significantly different from “0” (one sample t-test, t(7) = 4.94, p<.005), there were no differences across hand or target/distractor conditions. Specifically, the results of a 2 (Hand) by 4 (Condition) repeated measures ANOVA on the z scores did not reveal any significant effects – main effect for Hand, F(1,7) = 1.04, p>.34; main effect of Condition, F(3, 21) = 1.21, p>.36; and the Hand by Condition interaction, F(3, 21) = 1.04, p>.82. These results suggest that the specific effect of Hand in MT was not result of a limb-specific trade-off between RT and MT.

## Discussion

The present study investigated the patterns of distractor interference that emerge when participants have to select between target and non-target responses within a limb hemisphere system and between limb-hemisphere systems. The pattern of distractor interference that emerged can be described as a contralateral effect. Essentially, whenever distractors activated a competing response for the limb that was not responding to the target (i.e., distractor was contralateral to the responding limb), RTs were longer than when distractors activated a competing response for the limb that was responding to the target. This finding contrasts the ipsilateral effect that can occur in single limb distractor interference studies [3], [8], [11], and suggests that selection between target and non-target responses activated for the different limbs requires more time and effort to resolve than selection between responses for the same limb (see Rosenbaum [14]). More time and effort may have been required for between-limb selection because the response competition needed to be resolved prior to movement onset, whereas within-limb selection could be completed online (see also [7], [15]).

The contralateral effect is a novel addition to the body of literature that helps us understand the interplay between action and attention. Previous studies have shown that distractors that were ipsilateral and/or are closer to the responding hand caused the greatest amount of interference [3], [8]. There are, however, also studies that indicate that the distance of the distractor to the hand is not the only factor to influence patterns of distractor interference. For instance, Meegan and Tipper [16] showed that distractors that are close to the hand, but are more complex to execute, cause less interference than distractors that are farther away and simpler to execute. In a further exploration of the role of response efficiency on distractor interference, Welsh and Zbinden [10] found that distractors that have a higher index of difficulty cause less interference than distractors with a lower index of difficulty at a given spatial location. Taken together, these studies seem to indicate that distractor interference is based on the efficiency of the planning and execution of competing responses. The findings from the present study suggest that limb selection is another important factor, in addition to response efficiency, that influences patterns of distractor interference.

If response efficiency was the only factor taken into account during response competition, then distractors within a limb would have caused more interference than distractors for the other limb. In contrast, our results show that distractors that activated competing responses for the other limb were the only distractors to cause interference. This unique finding suggests that there are other factors related to response planning and execution that influence the interaction of action and attention. Therefore, it is critical to explore what unique response planning constraints limb selection places on the interconnected action and attention systems. First, previous studies on action centered attention have utilized tasks where limb selection is already known prior to stimulus presentation. Removing limb selection from response planning eliminates a process that has been shown to have the greatest cost on RT when not known in advance [14]. Second, competing responses within a limb will vary in direction and amplitude; therefore, response competition does not have to be completely resolved prior to response execution. The following paragraphs will address both of these topics: starting with the RT cost of limb selection, followed by how differences in planning and control might influence patterns of distractor interference.

The main finding from the present study is consistent with research by Rosenbaum [14] that revealed that there are larger increases in RT in a choice RT movement task when there is uncertainty regarding limb selection in comparison to conditions where movement direction and amplitude are unknown. This finding provides evidence that limb selection is time intensive and is likely to occur early in response selection and planning. Therefore, it is possible that the contralateral effect is largely attributed to the fact that the competition between responses for opposing limbs takes longer to resolve than competition between responses for the same limb leading to an increased RT cost.

Another, perhaps related, factor that may contribute to the increased RT during response competition is that this time intensive process must be completely resolved prior to response execution. The time and energy cost of initiating a movement with one limb, inhibiting it, and then initiating a movement with the other limb is likely to be greater than spending more time during the motor preparation period to ensure that the correct response is initiated. In contrast, when limb use is predetermined, the most time intensive response planning process has been eliminated and response competition, for distractors that code for difference directions and amplitudes, can be resolved online. The evidence that response conflict is not always resolved prior to response initiation comes from studies that demonstrate that movement trajectories are altered when movements are made in the presence of distractors [6], [11], [12]. In summary, it appears that patterns of distractor interference will be influenced by the interaction of: 1) the time required to plan the various components of a response; and, 2) whether the response conflict needs to be resolved prior to response execution.

An unexpected finding was the reduction in MT for the left hand when the distractor was in the opposite side of space. It is unlikely that this MT difference reflects a RT and MT trade-off due to the fact that it was only seen in the left hand. If this was a general strategy that individuals utilized, then we would expect to see RT traded for MT across all conditions and for both hands. Instead, there was no clear pattern of reliable differences. A more likely explanation is that this MT difference is due to hemispheric differences in (right hemisphere-left hand system dominant) attentional resources or (left hemisphere-right hand system dominant) response planning and execution, but we hesitate to speculate too much on this point as there is no clear explanation for this inconsistent pattern of effects.

In conclusion, the present study adds to the growing body of literature on action centered attention. The novel finding in the current study is that during a choice limb task, distractors that activate competing responses for the opposite limb cause more interference than within-limb distractors. Future research could further explore how space may modulate hand centered attention. For example, it might be interesting to assess interference effects in split brain patients who have increased independence of the limb-hemisphere systems.
